# Agar Composition Modulates Production of *Trichoderma* Peptaibols, Affecting Antibacterial and Antiproliferative Activity

**DOI:** 10.1007/s00284-025-04322-x

**Published:** 2025-06-20

**Authors:** Patrik Macášek, Adriana Kapustová, Jitka Viktorová, Ján Víglaš, Petra Olejníková, Lukas Grey, Lucia Laubertová, Helena Gbelcová

**Affiliations:** 1 Faculty of Medicine, Institute of Medical Biology, Genetics and Clinical Genetics, Comenius University, Sasinkova 4, 811 08 Bratislava, Slovak Republic; 2 Department of Biochemistry and Microbiology, University of Chemistry and Technology Prague, 166 28, Prague, Czech Republic; 3 Faculty of Food and Chemical Technology, Institute of Biochemistry and Microbiology, Slovak University of Technology in Bratislava, 812 37 Bratislava, Slovak Republic; 4 Faculty of Medicine, Institute of Medical Chemistry, Biochemistry and Clinical Biochemistry, Comenius University, Sasinkova 4, 811 08 Bratislava, Slovak Republic

## Abstract

Current antibiotics and chemotherapeutics are becoming ineffective because pathogenic bacteria and tumor cells have developed multiple drug resistance. Therefore, it is necessary to find new substances that can be used in treatment, either alone or as sensitizing molecules in combination with existing drugs. Peptaibols are bioactive, membrane-active peptides of non-ribosomal origin, mainly produced by filamentous fungi such as *Trichoderma* spp. This study focused on producing peptaibol-rich extracts from *Trichoderma atroviride* O1, cultivated on malt extract agar (MA) under circadian and constant darkness conditions for 13 days. Peptaibol production was detected by MALDI-TOF mass spectrometry after six days of cultivation. The extracts demonstrated antibacterial activity against *Staphylococcus aureus* strains, particularly the methicillin-resistant variant, but not against the Gram-negative *Pseudomonas aeruginosa*. Quorum sensing interference revealed that a peptaibol-rich extract suppressed *Vibrio campbellii* BAA-1119’s AI-2 signaling system to a degree comparable with gentamycin. Beyond antibacterial properties, the extracts exhibited notable antiproliferative activity against human ovarian cancer cells and their adriamycin-resistant subline in both 2D and 3D models. Specifically, MA-derived extracts reduced ovarian cancer cell viability by 70% at 50 μg/mL, especially under light/dark regime of cultivation. Compared to previously published results for PDA-based extracts, MA cultivation shifted the biological effects of peptaibol-containing extracts toward anticancer potential. These findings support the idea that modifying fungal cultivation parameters, the bioactivity of secondary metabolite mixtures can be tailored for specific therapeutic applications.

## Introduction

The constantly increasing number of oncological diseases is a serious medical and social problem. Chemotherapeutic resistance has been reported for almost all the drugs used to treat the most lethal cancers [1–3]. Resistance to adriamycin [4, 5], paclitaxel [6, 7], 5-fluorouracil [8, 9], cyclophosphamide [10], cisplatin [7, 11–14], carboplatin [15, 16], oxaliplatin [17–19], docetaxel [5, 20], and irinotecan [21] strongly suggests that the chemoresistance phenomenon is a major threat to the health and survival of cancer patients. Approximately 80–90% of mortality in cancer patients is directly or indirectly attributable to drug resistance [22]. In addition, there is increasing evidence from preclinical and clinical studies of the presence of tumor type-specific intratumoral bacteria [23]. The treatment of bacterial infections has also become much more complicated in recent years. In 2019, bacterial resistance directly caused 1.27 million deaths and contributed to 4.95 million globally [24]. The long-term use of highly effective antibiotics in the past has led to the emergence of multiresistant strains of bacteria that are capable of biofilm formation and quorum sensing, which are the most serious problems [25].

Attention is therefore focused on the search for new drugs capable of preventing or slowing the growth and division of cancer cells and/or pathogenic bacteria that have developed resistance. These are substances capable of acting directly on cancer cells and/or pathogenic bacteria or acting in combination with other drugs, as adjuvant substances sensitizing molecules in combination with existing drugs. Secondary metabolites produced by filamentous fungi represent an important group of biologically active compounds with potential applications in agriculture, medicine, and biotechnology. Among them, peptaibols are a unique class of membrane-active peptides with a length of 5–20 amino acid residues (500–2100 Da) [26, 27], characterized by the presence of non-standard amino acids such as α-aminoisobutyric acid (Aib) and an N-terminal acetyl group [28]. These compounds are synthesized by non-ribosomal peptide synthetases (NRPS) and have been primarily isolated from members of the *Trichoderma* genus. The biological activities of peptaibols include antimicrobial, antifungal, cytotoxic, and immunomodulatory effects. Their amphipathic structures enable them to interact with biological membranes, leading to pore formation and membrane disruption [29]. Various environmental and cultivation factors, such as the composition of the growth medium, temperature, pH, and light conditions, are known to influence peptaibol production [30]. Previous studies have indicated that the use of different cultivation media can modulate both the quantity and the spectrum of secondary metabolites produced [31]. However, information on how medium composition specifically affects the biological profile of peptaibol-rich extracts, particularly in terms of antiproliferative versus antibacterial activity, remains limited.

In this study, we investigated the production of peptaibol-containing extracts from *T. atroviride* O1 cultivated on malt extract agar (MA) and evaluated their antibacterial and antiproliferative activities. We aimed to assess how cultivation conditions, especially the choice of medium and incubation time, impact the bioactivity of the resulting extracts.

## Materials and Methods

### Cultivation and Extraction of Metabolites

The extracts of peptaibols were prepared and characterized according to the procedure developed at the Institute of Biochemistry and Microbiology, Faculty of Food and Chemical Technology, Slovak Technical University in Bratislava as described by Víglaš et al. In brief, peptaibols were produced by *Trichoderma atroviride* strain O1 [28].

### Fungal Strain and Culture Conditions

*Trichoderma atroviride* O1 was used in this study. The strain was maintained on potato dextrose agar (PDA; Sigma-Aldrich) at 4 °C and subcultured every 4 weeks to ensure viability.

#### Pre-cultivation Step

The pre-cultivation step was crucial for obtaining healthy and physiologically active mycelium for inoculation. *T. atroviride* O1 was pre-cultivated on PDA plates at 21 °C for 3 days. A 5 mm mycelial plug was then transferred to fresh PDA and subcultured twice, each time for two days, under either daylight conditions (DL; natural daylight ~ 10 h, November, Central Europe, 2019) or constant darkness (D).

#### Cultivation and Metabolite Production

A 5 mm mycelial plug from an actively growing colony was inoculated onto malt extract agar (MA; Sigma-Aldrich) poured into sterile Petri dishes (10 cm diameter, containing 25 mL of MA) lined with sterile cellophane. The cultures were incubated at 21 °C for up to 13 days under DL or D conditions. Growing mycelia were collected at various time points, frozen in liquid nitrogen, and ground into a fine powder.

#### Extraction of Metabolites

0.3 g of powdered mycelium was extracted in 1 mL of 60% ethanol (Mikrochem, Slovakia). The extraction procedure was tree times repeated - 1 min vortexing followed by 5 min incubation at the room temperature. After extraction, the mycelial debris was removed by centrifugation at 19.000× *g* for 5 min. The resulting supernatant was collected as the ethanolic extract and stored at − 20 °C.

The presence of peptaibols in the extracts was verified by MALDI-TOF mass spectrometry following the protocol described by Víglaš et al. [28]. Extracts obtained on days 3, 6, 8, 10, and 13 of cultivation were subsequently tested for their biological activity.

### Cell Lines Culturing

Human ovarian cancer cells HOC (A2780, Sigma-Aldrich), human ovarian cancer cells resistant to adriamycin ADR (A2780ADR, Sigma-Aldrich) were cultured in Dulbecco’s Modified Eagle’s Medium (DMEM, Sigma-Aldrich, D6429) supplemented with 10% fetal bovine serum (FBS, Sigma-Aldrich, F7524) and antibiotic antimycotic solution (Sigma-Aldrich, A5955). Adriamycin (Sigma-Aldrich, D5220) was added to the culture medium at the final concentration of 0.08 µM to maintain the resistance of the ADR cells. Human renal tubular epithelial cells RPTEC (CHT-003-0002, Evercyte) were cultured in DMEM containing 5% FBS. All the cell lines were cultured in 5% humidified CO_2_ atmosphere at 37 °C.

### Automated Screening of Peptaibol-Containing Extracts for Antiproliferative Activity in Vitro

The amount of 1 × 10^4^ cells/well of 96-well plates was inoculated into individual wells using the BioTek 405 TS Microplate Dispenser (Agilent). After 24 h post inoculation, the cells were washed with PBS solution and 100 μL of fresh DMEM medium with the tested extracts of peptaibols was added to a final concentration range of 0.625–10 μL/mL. The cells were incubated in the presence of the peptaibol-containing extracts for further 72 h. Finally, the cells were incubated with resazurin (0.03 mg/mL in PBS, Abcam) for 2 h. Fluorescence (560/590 nm, ex./em.) was measured using SpectraMax i3x Multi-Mode Microplate Reader (Molecular Devices).

Relative compound activity (RA) was determined as a percentage according to an equation:

$$RA\left( {\text{\% }} \right) = 100 \times \frac{{{\text{sample fluorescence}} - {\text{average fluorescence of NC}}}}{{{\text{average fluorescence of PC}} - {\text{average fluorescence of NC}}}}$$

Being NC the negative control and PC the positive control. The concentration of peptaibol-containing extracts that causes a 50% decrease in the number of viable cells compared to control cells (IC_50_ values) for individual peptaibol-containing extracts was calculated using the online tool freely provided by ATT Bioquest-IC50 calculator (https://www.aatbio.com/tools/ic50-calculator).

The selectivity index (SI) was determined as the ratio of IC_50_ value for non-cancerous cells to the IC_50_ value for cancer cells. For resistant cell lines, the collateral sensitivity (CS) was expressed as the ratio of the sensitive cancer cells IC_50_ value to the resistant cancer cells IC_50_ value. Selectivity is considered as “strongly selective” if the SI or CS value is greater than 6, “moderately selective “ if the SI or CS value is between 3 and 6, “slightly selective” if the SI or CS value is between 1 and 3 and “non-selective” if the SI or CS value is less than 1 [32].

### Sensitization of ADR Cells by the Selected Peptaibol-Containing Extracts

Adriamycin-resistant cells (ADR) were inoculated into 96-well plates in amount of 1 × 10^5^ cells/mL. After 24 h post inoculation, the cells were washed with PBS and fresh DMEM supplemented with the tested compounds at the final concentration of IC_25_ value and adriamycin in the concentration range 1–20 µM was added. The resazurin assay was used to determine cells viability after 72 h.

### The Impact of Selected Peptaibol-Containing Extracts on Spheroids Formation

The U-shaped surface of 96-wells was covered with a microlayer of SeaKem LE Agarose (Lonza, Switzerland) enabling cells to form spheroids. The amounts of 1 × 10^4^ cells of individual cell lines in 100 μL of the corresponding culture medium were used for inoculation into the adjusted U-shaped 96-well plates. Tested peptaibol-containing extracts in the amounts of 0.625–10 μL/mL were added 24 h after inoculation. After additional 72 h, the effect of the peptaibol-containing extracts on the formation of spheroids was observed by Axio Vert. A1 (Zeiss) light microscope with Axiocam ICC 1 photodocumentation equipment and Axio Vision 4.8 software.

### Bacterial Strains

*Pseudomonas aeruginosa* (CCM 3855) and the multidrug resistant *P. aeruginosa* (NEM 986, [33]) were investigated as gram-negative bacterial strains in this study. The susceptibility of gram-positive bacteria was evaluated on *Staphylococcus aureus* (CCM 3935) and on *Staphylococcus aureus* L12 (clinical isolate from central venous catheter, resistant to methicillin, erythromycin and ciprofloxacin). Two *Vibrio campbellii* strains, BAA-1118 and BAA-1119 (ATCC), were used to test quorum sensing.

The microorganisms were obtained from the Collection of Laboratory of Medical Microbiology (NEM, Czech Laboratory, Ltd., Prague, Czech Republic), Czech Collection of Microorganisms (CCM), Masaryk University, Brno and the American Type Culture Collection (ATCC) and were cultured in suspension in Mueller Hinton Broth (MHB, Sigma-Aldrich, 70192) at a temperature of 37 °C and a pH of 7.4.

### Antibacterial Activity of Peptaibol-Containing Extracts in Vitro

First, the antibacterial activity of peptaibol-containing extracts was screened against sensitive and multiresistant strains of *Pseudomonas aeruginosa*. A cell suspension with an optical density value of 0.5 was diluted 300 times (according to ISO standard 20776-1) and inoculated into a 96-well plate. After 24 h of cultivation, bacterial cells were affected by peptaibol-containing extracts at a final concentration of 1%. The antibacterial effect of peptaibol-containing extracts was evaluated after a further 24 h of cultivation using the resazurin assay.

The antibacterial activity of peptaibol-containing extracts on both *S. aureus* strains was evaluated by the broth microdilution method. The overnight inoculum of *S. aureus, S. aureus* L12 was adjusted on McFarland 0.5 with the fresh MHB. Then 10 µL of peptaibol-containing extracts (diluted 10, 20 and 40 times) resulting in final extract concentrations of 12.5–50 µL/mL were placed in the 96-well plate and the 190 µL of bacterial inoculum (0.5 McFarland) in MHB was added. Bacterial growth was observed based on turbidity, that was measured spectrophotometrically (A_630_) during the cultivation under shaking (250 rpm), 37 °C, 8–10 h. The growth was compared with the control containing 7% ethanol (solvent). The experiment was carried out in triplicate and stopped when the control reached the stationary phase.

### Inhibition of Quorum Sensing

The overnight culture of *V. campbellii* strain was diluted in Autoinducer Bioassay medium (NaCl, 17.5 g/L; MgSO_4_ × 7 H_2_O, 12.3 g/L; casamino acids, 2 g/L) supplemented with 10 mM KH_2_PO_4_ (pH 7.0), 1 mM L–L to a concentration of 5 × 10^5^ CFU/mL. The prepared culture was plated into a white 96-well plate with a clear bottom, individual peptaibol-containing extracts were added in triplicate in the final concentration range of 0.625–10 μL/mL and then the plate was incubated for 8 h at room temperature. Penicillin (1–200 mg/L), clindamycin (5–500 mg/L), gentamicin (2–300 mg/L), cefotaxime (0.5–100 mg/L), kanamycin (5–1000 mg/L), tetracycline (0.5–50 mg/L), erythromycin (1–200 mg/L), vancomycin (0.5–100 mg/L), spectinomycin (1–200 mg/L), ofloxacin (0.5–100 mg/L), ampicillin (0.5–100 mg/L), chloramphenicol (0.25–100 mg/L), oxacillin (1–200 mg/L), ciprofloxacin (1–50 mg/L) and sulfamethoxazole (0.5–50 mg/L) were used as positive controls. Then, luminescence was measured every 20 min (integration time 10,000 ms; shaken for 60 s prior to measurement) for 16 h in a microplate reader (SpectraMax i3x Multi-Mode Microplate Reader) at 30 °C. The EC_50_ (the sample concentration that halved the cell communication) of the compounds was determined from the sum of the luminescence. The viability of the culture was then checked by the resazurin assay, and the IC_50_ values of the compounds were determined. Compounds were compared based on EC_50_ and IC_50_, which were calculated by using GraphPad Prism software version 5.00 for Windows with non-linear regression curve fitting (GraphPad Software, San Diego, USA). All samples were run in triplicates.

## Results

### MALDI—TOF Monitoring of Obtained Peptaibol-Containing Extracts

**Fig. 1:**
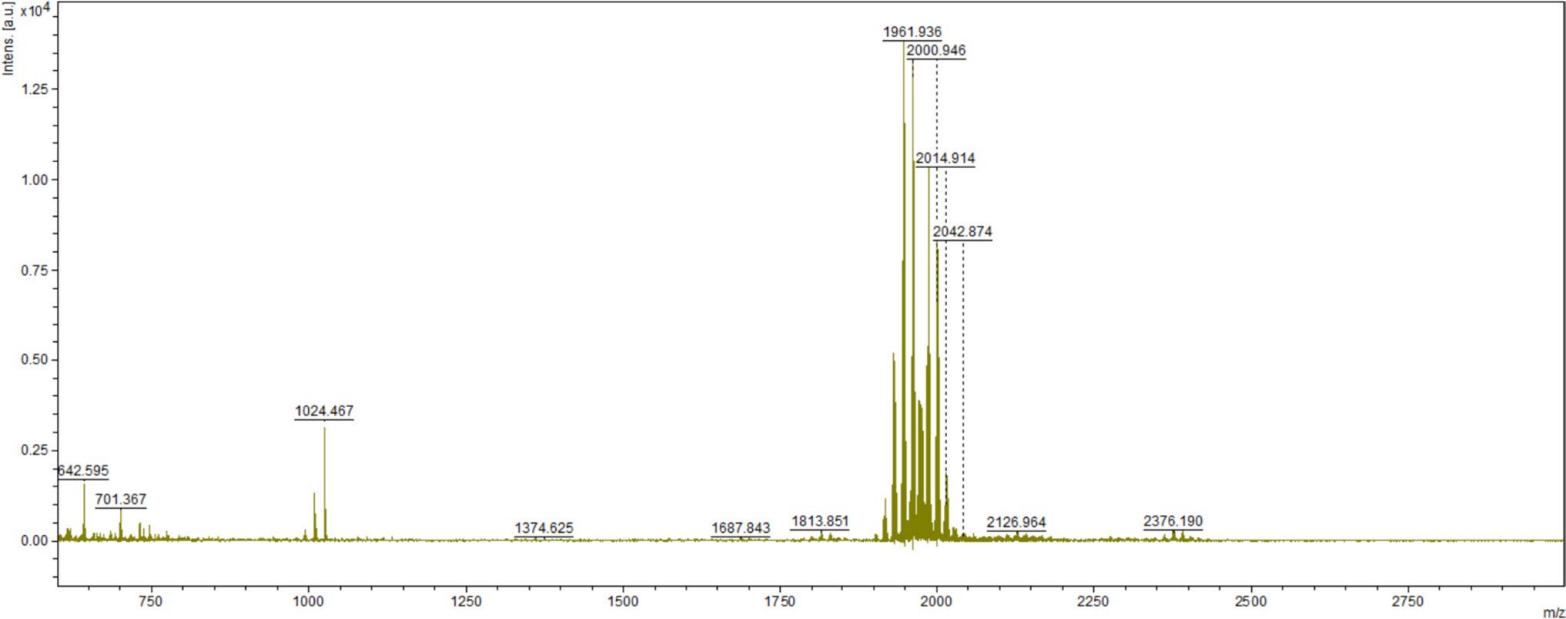
MALDI-TOF spectrum of peptides isolated from the mycelium of *Trichoderma atroviride* O1 after 10 days of cultivation. Peptaibols typical of *T. atroviride* are of *m/z* between 1900 and 2000

Peptaibol production by *Trichoderma atroviride* O1 was successfully confirmed using MALDI-TOF mass spectrometry. The characteristic peptaibol mass peaks were detected in extracts collected at various cultivation time points. The peptaibol signal intensity increased with prolonged cultivation time, indicating the accumulation of peptaibols in the medium as the culture transitioned into the stationary growth phase. We detected the presence of peptides with *m/z* 1900–2000 corresponding to peptaibols produced by *T. atroviride* (Fig. 1), described as atroviridins [28].

Due to the general nature of the isolation method, the spectrum shows the presence of unique peptide metabolites. The *m/z* of the peaks obtained overlap closely with the peaks detected in Víglaš et al. [28]. However, the shorter masses are different, suggesting also differences in metabolite production, which may be a consequence of the different agar used (MA).

### Antiproliferative Activity of Peptaibol-Containing Extracts on Sensitive and Resistant Ovarian Cancer Cells in Vitro

To study the antiproliferative effect of the MA peptaibol-containing extracts, a set of cancer cells, specifically ovarian cancer cells (HOC) and its adriamycin-resistant subline (ADR) and non-cancerous human renal tubular epithelial cells (RPTEC), were used to determine the selectivity index (SI) and collateral sensitivity index (CS) (Table 1).

**Table 1:** Cytotoxic potential of peptaibol-containing extracts against adriamycin-sensitive and adriamycin-resistant human ovarian cancer cells

	IC_50_ [µL/mL]			CS	SI	
PCE	RPTEC	HOC	ADR	HOC/ADR	RPTEC/HOC	RPTEC/ADR
6 d LD PDA^a^	˃ 10	5.9 ± 2.0	˃ 10	–	**˃ 1**	–
6 d D PDA^a^	˃ 10	˃ 10	˃ 10	–	–	–
6 d LD MA	4.5 ± 0.4	2.8 ± 0.2	4.7 ± 1.4	0.6 ± 0.2	**1.6 ± 0.2**	1.0 ± 0.4
6 d D MA	˃ 10	3.5 ± 0.7	˃ 10		**˃ 1**	
8 d LD PDA^a^	˃ 10	5.6 ± 0.6	5.5 ± 0.6	1.0 ± 0.2	**˃ 1**	**˃ 1**
8 d D PDA^a^	˃ 10	˃ 10	˃ 10	–	–	–
8 d LD MA	3.6 ± 1.5	3.6 ± 0.1	3.5 ± 1.5	1.0 ± 0.5	1.0 ± 0.4	1.0 ± 0.9
8 d D MA	4.4 ± 1.3	4.0 ± 0.0	3.2 ± 1.4	1.2 ± 0.6	1.1 ± 0.3	1.4 ± 1.0
10 d LD PDA^a^	˃ 10	4.2 ± 0.0	5.0 ± 0.6	0.8 ± 0.1	**˃ 1**	**˃ 1**
10 d D PDA^a^	˃ 10	˃ 10	˃ 10	–	–	–
10 d LD MA	3.4 ± 0.7	4.0 ± 0.0	3.5 ± 1.2	**1.6 ± 0.4**	0.9 ± 0.2	1.0 ± 0.5
10 d D MA	4.2 ± 0.1	4.5 ± 0.6	3.3 ± 1.3	1.4 ± 0.7	0.9 ± 0.1	1.3 ± 0.5
13 d LD PDA^a^	5.7 ± 0.8	3.3 ± 1.6	4.6 ± 0.4	0.7 ± 0.4	1.8 ± 1.1	1.2 ± 0.3
13 d D PDA^a^	˃ 10	4.7 ± 0.5	˃ 10	–	**˃ 1**	**˃ 1**
13 d LD MA	4.1 ± 0.1	1.4 ± 0.6	2.9 ± 1.1	0.5 ± 0.4	3.0 ± 1.4	1.4 ± 0.6
13 d D MA	4.3 ± 0.1	3.9 ± 0.0	3.4 ± 1.5	1.1 ± 0.5	1.1 ± 0.1	1.2 ± 0.6

The tested peptaibol-containing extracts (PCE) were obtained from a colony of *T. atroviride* O1 on different days of cultivation (X d, d = day) in the dark mode (D) or in the circadian rhythm (LD) on MA or PDA agar [28]; the exposure time to the compounds was 72 h. Experimental models—human renal tubular epithelial cells (RPTEC), human ovarian cancer cell line (HOC) and its adriamycin-resistant subline (ADR). Data are expressed as the average concentration that halved cell viability (IC_50_), the collateral sensitivity index (CS) and selectivity index (SI) of three replicates with standard error of the mean (SEM). Selectivity is considered as strongly selective, when CS or SI value is greater than 6, moderately selective when 6 ˃ CS or SI ˃ 3, slightly selective when 3 ˃ CS or SI ˃ 1 and non-selective when CS or SI value is less than 1. Significant values are shown in bold

^a^data obtained from the previous published paper [28]

To compare the efficacy of the antiproliferative activity of the extracts obtained by both cultivation methods, on PDA and MA, both indexes (CS, SI) were also calculated for the PDA peptaibol-containing extracts (Table 1), whose screening for antiproliferative activities was previously published by Víglaš et al*.* [28].

Extracts obtained on the third day of the cultivation of *T. atroviride* O1 on PDA or MA agar, in the dark (D) or during the light–dark (LD) rhythm had no effect on cell proliferation. In general, the peptaibol-containing extracts obtained from MA cultivation were more effective than those obtained from PDA cultivation (Table 1). They showed a higher antiproliferative potential against adriamycin-sensitive cells compared to the adriamycin-resistant ovarian cancer cells, the slight collateral sensitivity was observed in the case of extract after 10 days LD MA cultivation.

Almost all PDA extracts had an IC_50_ value higher than 10 µL/mL, showing at least a slight selectivity against ovarian cancer cells. In contrast, only two of the tested MA extracts tested, obtained after 6 days of cultivation, showed slight selectivity against ovarian cancer cells, 6 days LD MA and 6 days D MA (Table 1).

### Effect of Peptaibol-Containing Extracts on Spheroid Formation

As previously published, from the extracts produced by *T. atroviride* O1 on PDA, the effect of 10 days LD and 10 days D on the formation of spheroids composed of HOC or ADR cells in vitro was tested [28]. To maintain the experimental conditions, the same extracts produced by *T. atroviride* O1 on MA were selected to study their influence on the 3D experimental model.

**Fig. 2:**
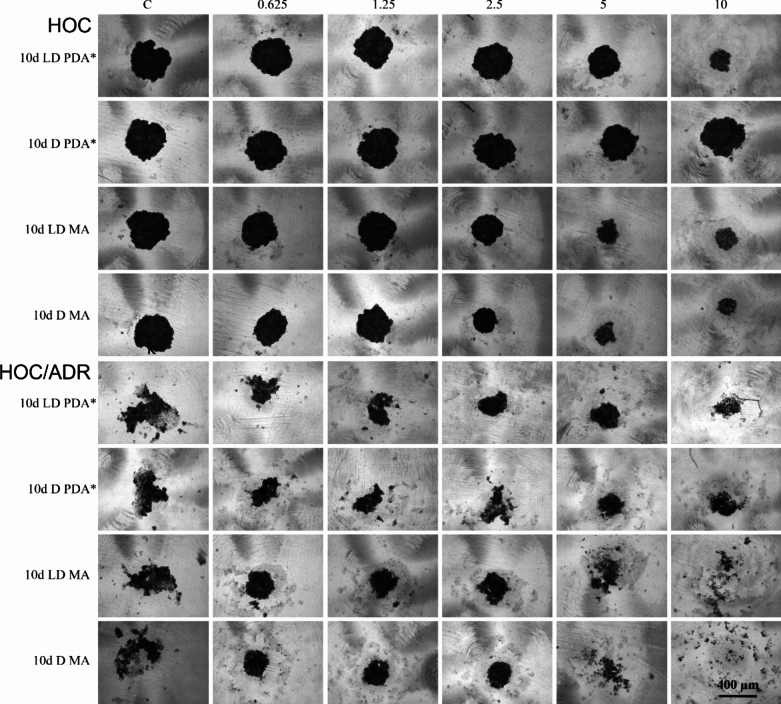
Effect of peptaibol-containing extracts on spheroid formation

HOC cells formed compact spheroids and were therefore used to evaluate the extracts. As shown in Fig. 2, the tested extracts caused a concentration-dependent inhibition of their formation. Extracts obtained from MA cultivation inhibited spheroid formation of HOC cells more than extracts from PDA cultivation (Fig. 2). ADR cells did not form compact spheroids. However, extracts from PDA cultivation slightly supported their formation, while the effect was already observed at the lowest concentration tested and was constant with increasing concentration (Fig. 2). Interestingly, extracts from MA culture significantly promoted spheroid formation at three lower concentrations tested (0.625; 1.25 and 2.5 μL/mL) and completely eliminated spheroid formation at two higher concentrations tested (5 and 10 μL/mL) (Fig. 2).

Adriamycin-sensitive (HOC) and adriamycin-resistant (ADR) human ovarian cancer cells were used as experimental models. The tested peptaibol-containing extracts were obtained on the 10th day of *T. atroviride* cultivation under circadian rhythm (10 days LD) or in the dark (10 days D) on MA and are compared with previously published results on extracts obtained from the cultivation on PDA, marked with * [28]. The amount of extracts applied ranged from 0.625 to 10 µL per 1 mL of culture medium. The time of peptaibol-containing extracts action—72 h, photodocumentation—Zeiss Axio Verte.A1, Axiocam Icc 1.

### Sensitization of ADR Cells by the Selected Peptaibol-Containing Extracts

As the 10 days D MA extract was the most potent of those tested in the 3D setup, it was further tested for its ability to sensitize the ADR cells to the effect of adriamycin. The peptaibol-containing extracts were used at a final concentration equal to their IC_25_ value and adriamycin at a concentration range of 1–20 µM. Sensitization potential was determined as the ratio of the IC_50_ of adriamycin to the IC_50_ of adriamycin affected by the peptaibol-containing extract at a concentration corresponding to its IC_25_ value. A fold change greater than 1 indicated a synergistic effect of adriamycin and the tested peptaibol-containing extract (Table 2).

**Table 2:** Sensitization potential of selected peptaibol-containing extract against the adriamycin-resistant human ovarian cancer cell HOC/ADR

	IC_25_ of PCE [µL/mL]	IC_50_ of adriamycin [µM]	Fold change
PCE 10 d D MA	3.3	2.1 ± 0.2	1.2 ± 0.1
Adriamycin	–	2.5 ± 0.0	–

Peptaibol-containing extract (PCE) was obtained from a colony of *T. atroviride* O1 on the 10th day of cultivation (10d) in dark mode (D) on MA; the exposure time to PCE was 72 h. Selected PCE was applied at a final concentration equal to its IC_25_ value and adriamycin in a concentration range of 1–20 µM. Fold change was determined as the ratio of the IC_50_ of adriamycin to the IC_50_ of adriamycin affected by PCE at the concentration corresponding to its IC_25_ value. Data are expressed as mean inhibitory concentration (IC_50_) or fold change of three replicates with standard error of the mean (SEM)

### Antibacterial Activity of Peptaibol-containing Extracts on Bacterial Strains in Vitro

The antibacterial activity of the peptaibol-containing extracts was evaluated against both antibiotic-sensitive and antibiotic-resistant strains of *S. aureus* and *P. aeruginosa*. Extracts obtained after longer cultivation times (13 days) in LD conditions exhibited enhanced antibacterial effects. However, compared to previous results obtained with PDA-derived [28] extracts, the antibacterial activity of MA-derived extracts was relatively lower, indicating a shift in the bioactivity profile. The higher effect was observed against the antibiotic-resistant strain *S. aureus* L12, which was completely inhibited in the presence of 5 µL/mL of the extract. In contrast, the growth of the antibiotic-sensitive strain *S. aureus* CCM 3935 was inhibited up to 76% when treated with 50 µL/mL of the same extract (Table 3).

**Table 3:** The susceptibility of gram-positive *S. aureus* CCM 3935 (antibiotic-sensitive) and *S.aureus* L12 (antibiotic-resistant) strains in the presence of the only effective peptaibol-containing extract obtained after cultivation on malt agar

13 d LD MA	% Growth	
Concentration [µL/mL]	*S. aureus*	
	Antibiotic-sensitive strain	Antibiotic-resistant strain
Unaffected bacterial growth	100	100
50	24 ± 5	0
5	74 ± 7	0
2.5	95 ± 5	80 ± 5

The peptaibol-containing extract 13 d LD MA was obtained after 13 days (d) of cultivation of *Trichoderma atroviride* O1 in light–dark (LD) conditions on malt agar (MA). Exposure time was 8–10 h. Data are expressed as the mean of six replicates with standard error of the mean (SEM)

The peptaibol-containing extracts (PCEs) tested showed no antibacterial effect against the antibiotic-sensitive *P. aeruginosa* strain. On the contrary, almost all tested extracts appeared to stimulate the growth of this gram-negative bacterium (Table 4). These findings suggest that the metabolites produced preferentially inhibit gram-positive bacteria, as exemplified by the activity observed against *S. aureus*.

**Table 4:** The growth of gram-negative *P. aeruginosa* in the presence of peptaibol-containing extracts obtained from *Trichoderma atroviride* O1 cultivated on potato dextrose agar (PDA) or malt agar (MA)

PDA	% Growth		MA	% Growth	
	*P. aeruginosa*			*P. aeruginosa*	
	Sensitive	Resistant		Sensitive	Resistant
Unaffected bacterial growth	100	100	Unaffected bacterial growth	100	100
3 d LD	108 ± 0	75 ± 0	3 d LD	138 ± 3	112 ± 1
6 d LD	125 ± 1	84 ± 4	6 d LD	137 ± 4	138 ± 4
8 d LD	133 ± 6	103 ± 6	8 d LD	130 ± 6	122 ± 3
10 d LD	129 ± 3	126 ± 5	10 d LD	121 ± 0	123 ± 0
13 d LD	116 ± 1	102 ± 1	13 d LD	142 ± 7	127 ± 1
3 d D	115 ± 2	84 ± 1	3 d D	117 ± 3	63 ± 1
6 d D	122 ± 6	85 ± 1	6 d D	116 ± 2	95 ± 2
8 d D	116 ± 3	90 ± 1	8 d D	113 ± 2	132 ± 11
10 d D	126 ± 1	101 ± 1	10 d D	119 ± 3	144 ± 0
13 d D	124 ± 2	92 ± 3	13 d D	119 ± 2	139 ± 2

*Trichoderma atroviride* O1 was cultivated in light–dark (LD) conditions or in constant darkness (D). The time of isolation of the peptaibol-containing extracts was on the 3rd, 6th, 8th, 10th and 13th day (d). The extracts were applied at 10 μL/mL. The exposure time to peptaibol-containing extracts was 24 h. Data are expressed as the average of six replicates with standard error of the mean (SEM)

MALDI-TOF analysis confirmed that peptaibols were undetectable or present at very low levels in extracts obtained after 3–6 days of cultivation. This confirms that peptaibol production by *T. atroviride* O1 is time-dependent, with measurable accumulation occurring only after longer cultivation periods (≥ 8 days), in accordance with [28]. Therefore, early extracts (3–6 days) represent peptaibol-deficient samples and effectively serve as internal controls to assess the potential biological activity of other Trichoderma-derived secondary metabolites. The biological effects observed in these early extracts are likely attributable to compounds produced during the early phase of fungal metabolism, independently of peptaibol synthesis. This approach allowed us to distinguish between peptaibol-related and peptaibol-independent bioactivity without the need for artificial manipulation of the extracts.

### Quorum Sensing Inhibition

Quorum sensing is one of the key systems used by bacteria for virulence. A reduction in the ability bacteria to communicate with each other can lead to a reduction in their virulence. To discriminate between the toxic and the quorum sensing inhibitory concentrations, the IC_50_ (concentration that halves viability) was compared with the EC_50_ (concentration that halves communication). If the dose of extract that induced toxicity was greater than the effective dose that reduced QS, the compounds tested were considered to be effective. The higher the IC_50_/EC_50_ ratio, known as the selectivity index (SI), the more potent the compound was in inhibiting QS. PCE 10 days D MA was selected for the quorum sensing inhibition assay as the most potent extract from the previous results. This extract was able to inhibit the bacterial communication (Table 5). The marine bacterium *Vibrio campbellii* naturally uses both autoinducer molecules—AI-1 and AI-2; however, to study a specific type of intracellular communication, the insertion-inactivation mutants were prepared. Based on our results, it appeared that peptaibol-containing extracts are more promising in inhibiting AI-2-based communication (Table 5), as mutant BAA-1119 is deficient in AI-1-based communication, whereas mutant BAA-1118 is deficient in AI-2 type of communication. The selectivity index is comparable to that of gentamicin which was found to be 1.53 for *Vibrio* BAA 1118.

**Table 5:** Anti-quorum sensing effects of selected peptaibol-containing extract on mutants of *Vibrio campbellii*

	*V. campbellii* BAA-1118			*V. campbellii* BAA-1119		
	IC_50_ [μL/mL]	EC_50_ [μL/mL]	SI [IC_50_/EC_50]_	IC_50_ [μL/mL]	EC_50_ [μL/mL]	SI [IC_50_/EC_50_]
10 d D MA	1.07 ± 0.03	0.64 ± 0.01	1.67	˃ 10	0.47 ± 0.06	˃ 21.27

*Trichoderma atroviride* O1 was cultivated in constant darkness (D) conditions on MA. The time of isolation of the peptaibol-containing extract was on the 10th day (d). The concentration range was 0.625–10 μL/mL. Exposure time was 16 h. Mutant BAA-1119 is deficient in AI-1 communication, mutant BAA-1118 is deficient in AI-2 communication. IC_50_—concentration that halves viability, EC_50_—concentration that halves communication, SI—selectivity index calculated as the ratio of IC_50_ and EC_50_

## Discussion

In recent years, the spread of resistance to chemotherapeutics [1] and antibiotics [24] has boomed. Therefore, alternatives in the form of new drugs or chemoadjuvants are being intensively sought. An interesting group of natural compounds, showing the diversity of biological activities with potential applications in pharmacology and medicine, are the secondary metabolites of filamentous fungi [34]. From a chemical point of view, these compounds could be amino acids, anthracenones, butanolides, butenolides, cytochalasans, macrolides, naphthalenones, pyrones, terpenes, etc. [35].

Previously, we investigated the antiproliferative and antibacterial activity of the extracts containing the short non-ribosomal peptides produced by *Trichoderma atroviride* O1 when grown on PDA [28]. Since environmental and nutritional manipulations are known to have a substantial impact on the quantity and diversity of secondary metabolite production [36], we decided to investigate whether changing the agar from PDA to MA would enhance the previously described antiproliferative and antibacterial properties of peptaibol-containing extracts (PCE) produced by *T*. *atroviride* O1.

It appears, that the PCE obtained from MA cultivation were more effective compared to those from PDA cultivation, especially in the case of cultivation in the dark, when PDA extracts did not show significant antiproliferative activity on cancer cells in the concentration range tested, whereas MA did (Table 1, [28]). Although the synthesis of peptaibols is attributed to conidiation, which is stimulated by blue light [37], the presence of peptaibols was also detected in extracts from cultures of *T. atroviride* O1 grown in constant darkness [28]. However, the production was significantly delayed compared to light–dark conditions [28]. Therefore, it was surprising that the antiproliferative effects of PCE from MA cultivation in the dark were not only more significant compared to PCE from PDA cultivation, but also compared to MA cultivation in the light–dark rhythm when tested in 3D conditions (Fig. 2). The composition of nutrients in the culture medium, particularly the sources of carbon and nitrogen, plays a pivotal role in the production of secondary metabolites [31]. The growth media composition, the carbon source and nitrogen source, is related to the regulation and the expression of biosynthetic genes, including those encoding non-ribosomal peptide synthetases (NRPS) responsible for peptaibol production. For instance, in *Trichoderma asperellum*, the presence of high concentrations of glucose has been shown to repress NRPS gene expression, leading to reduced peptaibol synthesis. Conversely, utilizing sucrose as the carbon source alleviates this repression, resulting in enhanced peptaibol production. This modulation is attributed to glucose-sensing mechanisms that influence the transcriptional regulation of NRPS genes. This observation is consistent with earlier studies showing that the nutrient composition of the culture medium significantly affects the biosynthesis of secondary metabolites, including peptaibols. For example, variations in carbon or nitrogen sources and other physicochemical parameters are known to modulate the expression of non-ribosomal peptide synthetase (NRPS) genes involved in peptaibol biosynthesis [38]. These findings clearly indicated that optimizing the cultivation conditions is essential factor for achieving the desired composition and production of biologically active metabolites. These results suggest that MA supports the biosynthesis of metabolites with antiproliferative properties more effectively than PDA, which in earlier studies favored the production of antibacterial compounds [28]. Many drug candidates that show promise in vitro appear to be ineffective in vivo due to the high complexity and heterogeneity of different tumors. This is partly because the in vitro assays are based only on adherent cultures that do not reflect the complex microenvironment of solid tumors. Culturing mammalian cells in 3D models, called spheroids, should reliably represent the size-dependent microenvironmental changes such as cellular heterogeneity, hypoxic gradients and spatial distribution of necrotic and proliferating cells [39]. Therefore, we verified the antiproliferative activity of selected PCEs, which are active on HOC and ADR cells growing in 2D culture conditions on cells growing in the form of spheroids. In addition to confirming the potentiation of the antiproliferative activity of PCE from the MA cultivation compared to PDA cultivation, we observed a significantly different impact of PCE from the MA cultivation on resistant and sensitive cancer cells, while PCE from the PDA cultivation affected sensitive and resistant cancer cells in comparable manner (Fig. 2). However, sensitization of resistant ovarian cancer cells ADR grown in a monolayer to the effect of the chemotherapeutic agent adriamycin using the peptaibol-containing extract 10 days D MA was not successful. Therefore, we do not expect that the different antiproliferative effect of this extract on sensitive and resistant cells is the result of the impact of the extract on newly created mechanisms of resistance to adriamycin in resistant cells. Peptaibols exert their effects on resistant cell lines through several mechanisms, including membrane permeabilization, resistance to proteolysis, rapid cellular uptake, and the induction of cell death [28, 40, 41]. These mechanisms highlight the potential of peptaibols as effective agents in overcoming cellular resistance, thereby providing valuable insights into their therapeutic applications. Understanding these pathways is crucial for developing strategies that enhance the efficacy of peptaibols in combating resistant cell phenotypes.

In contrast to the antiproliferative effects of the PCEs on cancer cells, both antibiotic-sensitive and antibiotic-resistant *S. aureus* strains were affected only when treated with extracts obtained at the latest stage of cultivation (day 13) on malt agar (MA) under a light–dark (LD) regime. In our previously published study, however, extracts obtained from earlier cultivation stages of *T. atroviride* O1 on PDA, particularly under LD conditions, were also effective against both sensitive and resistant *S. aureus* strains, with the antibacterial activity increasing over time [28]. This trend was attributed to the gradual accumulation of peptaibols during extended cultivation [28].

These observations suggest that even in the case of antibacterial activity against gram-positive bacteria such as *S. aureus*, changing the growth medium from PDA to MA can negatively affect the production and efficacy of bioactive metabolites with antibacterial activity.

The growth of *P. aeruginosa* in the presence of PCEs was largely unaffected, with the strongest observed inhibition reaching only 25%, corresponding to 75% residual growth in the presence of extract from the 3rd day of PDA cultivation under LD conditions. At this stage, peptaibol production is only beginning [28], and the observed effect may result from synergistic interactions with other early-phase metabolites. Despite this limitation of PCEs toward gram-negative bacteria, we propose that peptaibols remain the principal active compounds responsible for the effects observed in this study—not only due to their confirmed presence in the extracts, but also because of their selective activity against gram-positive, but not gram-negative, bacteria. This observation is in line with known peptaibol properties and adds to the understanding of their spectrum of activity, particularly regarding resistant strains [42].

Finally, we tested the anti-quorum sensing activity of the selected PCE 10 days D MA, as inhibition of bacterial cell-to-cell communication has applications in the prevention and spread of bacterial infections. Communication is used by bacteria to sense their numbers and at certain breakpoints they switch their behavior and start to producing biofilm, thereby regulating their virulence and metabolism [43]. Nowadays, the quorum sensing modulators offer new tools in the fight against bacterial resistance and in the diagnosis of the disease, as well as acting as novel antibacterial agents [44]. For the practical application of the compounds as antibiotics, the SI value, which characterises the anti-quorum sensing activity of compounds should be higher than 10 [45]. This was not achieved in the case of the SI determined for the 10 days D MA extract for Vibrio BAA 1118, however the index is comparable to that of gentamicin determined for *Vibrio* BAA 1118.

In general, only a few publications deal with the anticancer activity of peptaibols [46–48] and little is known about their molecular action [28, 49]. For future studies of peptaibols, a more robust peptaibol production technique needs to be developed, e.g., by recombinant production of peptaibol synthase enzyme. Although *Trichoderma* spp. are well described producers of secondary metabolites, metabolite synthesis is susceptible to changes in fitness, including the duration of strain deposition. Secondary metabolites are generally required when the microorganisms are in contact with another competing organism, which is not usually the case in cultivation environments. However, the peptaibols and other secondary metabolites of fungi represent the promising candidates in the field of antibacterial and anticancer drugs or their adjuvants.

## Conclusions

The present study demonstrated that the cultivation conditions, particularly the growth medium (malt agar compared to potato dextrose agar), and the duration of cultivation, significantly influence the production and biological profile of peptaibol-containing extracts from *Trichoderma atroviride* O1.

Cultivation on malt extract agar led to the accumulation of metabolites with notable antiproliferative activity, reducing ovarian cancer cell viability by 70% at 50 μg/mL. In contrast, the previously reported cultivation on potato dextrose agar favored the production of metabolites with stronger antibacterial effects against gram-positive bacteria. Nevertheless, strong anti-quorum sensing potential was proved for extracts from malt agar with selectivity index comparable to that of gentamycin.

Our findings highlight the critical role of optimizing environmental and nutritional factors to tailor the biological activity of fungal secondary metabolites. These results also suggest that adjusting cultivation strategies can selectively enhance the production of bioactive compounds with potential applications in biomedical and biotechnological fields. To translate these findings into clinical practice, it is necessary to validate them in vivo and explore strategies for scale-up.
